# Variable hyperthyroidism outcomes related to different treatment regimens: an analysis of UK Biobank data

**DOI:** 10.1530/ETJ-24-0393

**Published:** 2025-04-14

**Authors:** Kris Elomaa, Matt Spick, Earn H Gan, Simon H Pearce, Nophar Geifman

**Affiliations:** ^1^ School of Health Sciences, Faculty of Health and Medical Sciences, University of Surrey, Guildford, UK; ^2^ Translational and Clinical Research Institute, International Centre for Life, Newcastle University, Newcastle upon Tyne, UK; ^3^ Department of Endocrinology, Translational and Clinical Research Institute, Newcastle University, Newcastle upon Tyne, UK

**Keywords:** hyperthyroidism, Graves’ disease, comorbidity, biomarkers, first-line, radiotherapy, thionamides, thyroidectomy

## Abstract

**Background:**

UK guidance on the assessment and management of thyroid disease was set out in NICE guideline NG145 in 2019 and is expected to result in an increase in radioactive iodine (RAI) being offered as a first-line definitive treatment for hyperthyroidism.

**Methodology:**

In this work we analyse longitudinal UK Biobank data to assess all-cause mortality and comorbidity risks associated with the main treatment modalities for 793 participants with hyperthyroidism, specifically antithyroid drugs (ATDs), RAI and thyroidectomy.

**Results:**

Participants treated with RAI showed reduced all-cause mortality compared with those treated with ATD alone (time to event ratio: 1.8, 95% CI: 0.9–3.6), albeit the result did not reach statistical significance, as did those treated by thyroidectomy (time ratio: 2.0, 95% CI: 1.1–3.9). For treated patients, odds ratios were generally elevated for osteoporosis, cardiovascular events and atrial fibrillation, but again did not reach statistical significance except for those patients treated by ATDs, with an odds ratio for atrial fibrillation of 2.2 (95% CI: 1.2–4.1) versus controls.

**Conclusion:**

Our findings were consistent with those previously reported in the literature and do not reveal any evidence from the UK Biobank to contradict the safety of RAI being offered as a first-line treatment. The data are also suggestive, however, that treatments do not fully eliminate risks of complications related to hyperthyroidism. This reinforces the need for both clear communication where there may be risks of complications such as osteoporosis as well as clinical support for patients even after definitive treatment.

## Introduction

Hyperthyroidism is a condition that occurs due to excess production and secretion of thyroid hormones by the thyroid gland (1). Eighty percent of cases are owing to Graves’ disease (GD; autoimmune hyperthyroidism) (2, 3); other causes include toxic multinodular goitre, toxic adenomas and medication-induced or idiopathic hyperthyroidism (4). GD has a lifetime risk of around 3% for women and 0.5% for men (5), with susceptibility thought to be driven by a mixture of genetic, epigenetic and environmental factors (6). Hyperthyroidism is usually treated by one of three approaches, all of which are long established (7). The first and most conservative of these are antithyroid drugs (ATDs), thionamides, which act as a preferential substrate for iodination, thus blocking production of the hormones triiodothyronine (T3) and thyroxine (T4). The second is radioactive iodine (RAI), I-131, which is preferentially taken up by – and destroys – thyroid tissues (8). While RAI is widely used for definitive treatment, it is contraindicated in cases of hyperthyroidism with severe orbitopathy or during pregnancy. The third established approach is thyroidectomy (9). Other approaches are emerging, such as radiofrequency ablation of the thyroid gland, but this has only recently been examined as a novel treatment for persistent or relapsed Graves’ disease (10).

It has long been recognised that chronic thyroid hormone excess can have long-term health consequences focused on the cardiovascular system and skeleton, which are sensitive to hyperthyroidism. This manifests as excess all-cause and cardiovascular mortality, increased atrial fibrillation and heart failure (11, 12). In addition, osteoporosis and reduced bone mineral density have been documented in hyperthyroid patients, providing another important reason for prompt and effective treatment (13).

UK guidance on the assessment and management of thyroid disease was set out in NICE guideline NG145 in 2019 (14). This guideline recommended that – for adults with Graves’ disease – RAI would be offered as the first-line definitive treatment unless ATDs were likely to achieve remission or radioiodine was unsuitable. Before this, common practice followed Royal College of Physicians guidance for ATDs to be employed as the first-line treatment, with radioiodine or thyroidectomy being considered in cases of persistent or relapsed hyperthyroidism (15). All NICE guidelines include rationale and impact sections, and NG145 based its conclusions on RAI producing better long-term outcomes than ATDs in terms of thyroid status as well as being cost effective; this choice is not currently shared by other countries or international societies. The rationale and impact section also stated that the ‘committee was aware that the recommendations would result in RAI being offered as first-line definitive treatment to more people than currently’ and also agreed ‘to make a research recommendation on the long-term effectiveness and safety of exposure to RAI’.

The UK Biobank is a global resource comprising anonymised genetic, proteomic, metabolomic and health information derived from clinical records and biological samples from over 500,000 UK participants (16). In this work we review UK Biobank data on individuals with hyperthyroidism, with a focus on outcomes, which have been associated with hyperthyroidism or its related treatments, including osteoporosis, atrial fibrillation and other cardiovascular events, as well as cancer mortality, which has been discussed in the context of secondary cancers induced by RAI (17, 18). We also review data from peripheral blood biochemistry for these populations. This is with the goal of assessing the advantages and disadvantages of different treatment regimens in a large observational cohort study, in order to contribute to the need for research on the long-term effectiveness and safety of RAI, as set out in NG145 (14).

## Materials and methods

### Study cohort and UK Biobank data

The UK Biobank covers information from around 0.5 million UK participants; the project was approved by the North West Multi-Centre Research Ethics Committee and all participants provided written informed consent. The study protocol is available online (https://www.ukbiobank.ac.uk).

Data were accessed (under application number: 83988) and pre-processed for this work as follows. Hyperthyroid participants were identified by their ICD10 (International Classification of Diseases, 10th revision) code E05. Participants were then categorised according to different treatment regimens using the codes set out in Table 1, which are based upon the OPCS Classification of Interventions and Procedures (19), the codes were provided by the UK Biobank in their Medical History interview data-field/GP clinical event records available through the UK Biobank. Diagnoses (ICD10-codes) for participants were identified using the UK Biobank’s ‘first occurrences of health outcomes’ and ‘hospital inpatient data’ fields (20, 21). Participants were included if the diagnosis was recorded in primary care or hospital inpatient records. All available diagnostic data sources in the UK Biobank include primary care and hospital inpatient data, national death registries and self-reported medical conditions from the UK Biobank baseline visit (https://www.ukbiobank.ac.uk/enable-your-research/about-our-data/health-related-outcomes-data).

**Table 1:** UK Biobank codes for hyperthyroidism treatment type included in this work. The first occurrence of the health variable combines diagnoses from hospital inpatient and primary care.

Condition	Source	Code(s)
Hyperthyroidism	The first occurrence of health outcomes	E05
	Hospital inpatient – summary diagnoses (ICD10-codes)	E05
ATDs	UK Biobank, baseline, self-reported medications	Propylthiouracil, propylthiouracil product, carbimazole
	GP prescription records	No data for ATDs
Radioiodine	UK Biobank, baseline verbal interview	1228
	OPCS-4	X655
	OPCS-3	998
	GP clinical event records (read codes)	5A16./Ub0wq/x02EA/XE1Rx
Thyroidectomy	UK Biobank, baseline verbal interview	1432
	OPCS-4	B08
	OPCS-3	070, 071, 072

**Figure 1:**
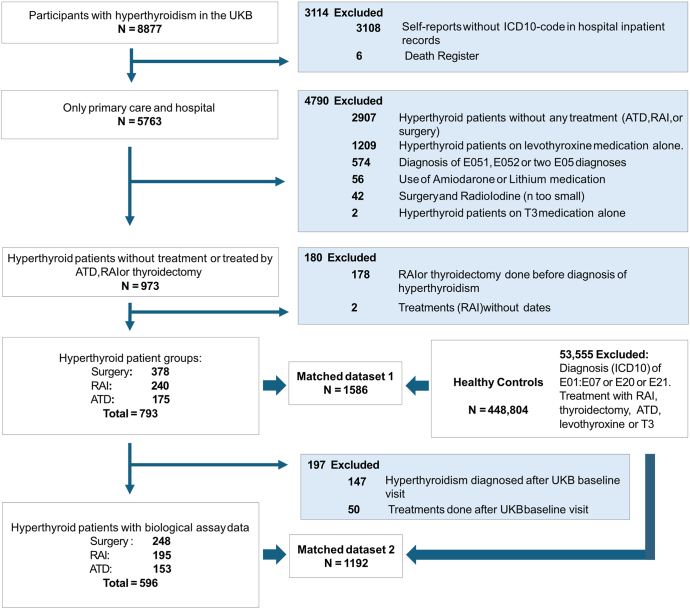
Flow chart of inclusions and exclusions from UK Biobank data.

A number of exclusion criteria were also applied. First, any death register cases or self-reports that were not verified by a diagnosis in hospital inpatient records were excluded (*n* = 3,114). Second, individuals using either amiodarone or lithium medications were excluded (*n* = 56). Third, individuals who were treated with both thyroidectomy and radioiodine were excluded due to the small sample size (*n* = 42). Fourth, individuals with a hyperthyroidism diagnosis but only treated with levothyroxine or triiodothyronine were excluded (*n* = 1,209). While hypothyroidism following other treatment (ATDs, RAI and thyroidectomy) for hyperthyroidism is commonly observed, treatment with levothyroxine only – without any prior treatment – raises the possibility that information on the patient’s treatment regimen is incomplete or incorrect. Similarly, individuals with a hyperthyroidism diagnosis but no record of treatment regimen were also excluded (*n* = 2,907). These individuals likely included a mixture of individuals with self-limiting hyperthyroidism or with incomplete information in the Biobank. Fifth, individuals coded as E05.1 or E05.2 were excluded, as these cover thyrotoxicosis with toxic multinodular goitre and thyrotoxicosis from ectopic thyroid tissue respectively, conditions with distinct aetiology from Graves’ disease. We also excluded cases where either thyroidectomy or RAI was performed before the diagnosis of hyperthyroidism (*n* = 178), due to risks of including treatments related to other thyroid conditions. Finally, we excluded cases where treatments did not include dates (*n* = 2). When examining the risk profiles between different managements for hyperthyroidism and subsequent complications (e.g. osteoporosis), we included only cases where both diagnosis of hyperthyroidism and the treatment date occurred before the diagnosis of the complication being assessed. Since data on medication dates were not available, we used the hyperthyroidism diagnosis date as the starting point for ATD medication. In total, we analysed 793 individuals with hyperthyroidism who were treated with surgery (*n* = 378), radioiodine (*n* = 240) or ATDs (*n* = 175). When analysing peripheral blood biomarkers, we further excluded 197 individuals from the cohort if they were diagnosed with hyperthyroidism or received treatment after the baseline Biobank assessment. This was done to ensure that the individuals had the condition and had been treated at the time of blood sampling. A flow diagram detailing the exclusions at each stage is set out in Fig. 1.

Controls were also identified and included in the analysis to create a baseline risk profile. Individuals with any thyroid- or parathyroid-related diagnoses were excluded. All cases of thyroid-related treatments were also excluded. We further excluded any cases where GP clinical event data detected use of radioiodine (I-131) or any antithyroid medication (propylthiouracil or carbimazole), levothyroxine or tri-iodothyronine, leaving us with a total pool of 448,804 controls. The MatchIt library in R was used to select a subset (*n* = 793) from the controls, matching for sex and age, using ‘exact’ matching for the sex variable and the nearest neighbour method for age.

### Statistical analysis

For all-cause mortality risk assessment, Schoenfeld residuals showed that the proportionality assumption did not hold, so an accelerated failure time (AFT) model using a Weibull distribution was constructed. This yielded time-to-event ratios, here defined as the ratio of time from treatment to mortality for one treatment type relative to another.

For the main clinically identified adverse health conditions associated with hyperthyroidism (atrial fibrillation, osteoporosis and a combined major cardiovascular event (MACE) endpoint, incorporating stroke, heart failure and myocardial infarction), adjusted odds ratios were calculated using a multivariable logistic regression model, where the relationship between treatment type and the condition was investigated, including age, sex, deprivation index, BMI, resting pulse rate and smoking status as covariates. Cancer diagnoses were also investigated (all cancers); cases of thyroid cancer (*n* = 5) were too few for statistical analysis.

In addition, peripheral blood biochemical analytes for individuals with hyperthyroidism (split by treatment regimen) and controls were also investigated for the subset of the population where these analytes were measured after treatment (22). Differences in the levels of these analytes were tested by Wilcoxon rank-sum test, with false discovery rate (FDR) correction by Benjamini–Hochberg. All statistical analyses were conducted in R (version 4.4.1), using the RStudio integrated development environment (version 2024.04.2) (23).

## Results

### Participant baseline characteristics

Cohort data from the UK Biobank participants with hyperthyroidism analysed in this work are summarised in Table 2. There were no statistically significant differences in the matched variables (age and sex) between controls and total cases of hyperthyroidism. For unmatched variables, individuals were more likely to be current smokers and had a higher level of deprivation. Deprivation at baseline is reported using the indices of multiple deprivation scores available in the UK Biobank dataset (24). The scores are based on the UK government’s qualitative studies of deprived areas within the local councils of England, Wales and Scotland, published at the time of the UK Biobank baseline visits (25).

**Table 2:** Baseline characteristics of the population. Mean ± SD are given for continuous variables. *P*-values were calculated using the Wilcoxon rank sum test (for continuous variables) and the chi-square test (for categorical variables). BMI and pulse rates are for the first measurement available after treatment.

Parameter	Controls	Hyperthyroidism	*P*-value
*n*	793	793	
Age	56.13 ± 8.11	56.13 ± 8.11	1
Males (%)	134 (16.9)	134 (16.9)	1
Deprivation	17.44 ± 13.89	18.68 ± 15.42	0.097
BMI	26.84 ± 4.88	27.96 ± 5.46	0.001
Resting pulse rate	65 ± 21	62 ± 24	0.008
Current smoker = yes (%)	69 (8.7)	113 (14.2)	0.001
Previous smoker = yes (%)	276 (34.8)	274 (34.6)	0.958

A summary breakdown of these characteristics by treatment type (ATD versus RAI versus thyroidectomy) is provided in Table 3. Those patients undergoing thyroidectomy were less likely to be male (14%) than the average for the cohort (17%). Patients with ongoing ATD treatment alone were more likely to be male (22%) and had the greatest prevalence of current smoking status, while patients treated by RAI had lower levels of deprivation. BMI and pulse rate (post-treatment) were slightly higher for the treated individuals than for controls. UK Biobank recruitment took place between 2006 and 2010 and it should be noted that the majority of the individuals analysed here had hyperthyroidism before this point (*n* = 596), so these data represent long-term outcomes rather than short-term follow-up measurements. Baseline UK Biobank measurement occurred at an average of 10.5 years (standard deviation 10.4 years) after treatment for these patients.

**Table 3:** Baseline characteristics for population. Mean ± SD are given for continuous variables. *P*-values were calculated using ANOVA or Kruskal–Wallis test (for continuous variables) and the chi-square test (for categorical variables). BMI and pulse rates are for the first measurement available after treatment.

Parameter	Controls	ATD	RAI	Surgery	*P*-value
*n*	793	175	240	378	
Age	56.13 ± 8.11	56.99 ± 7.92	56.67 ± 7.80	55.39 ± 8.35	0.104
Males (%)	134 (16.9)	39 (22.3)	43 (17.9)	52 (13.8)	0.092
Deprivation	17.44 ± 13.89	19.44 ± 15.34	16.88 ± 15.29	19.48 ± 15.48	0.049
BMI (mean (SD))	26.84 ± 4.88	27.81 ± 5.54	28.27 ± 5.98	27.82 ± 4.99	0.009
Resting pulse rate	65 ± 21	68 ± 17	59 ± 26	59 ± 25	<0.001
Current smoker = yes (%)	69 (8.7)	29 (16.6)	33 (13.8)	51 (13.5)	0.004
Previous smoker = yes (%)	276 (34.8)	57 (32.6)	83 (34.6)	134 (35.4)	0.93

### All-cause mortality: AFT analysis

**Figure 2:**
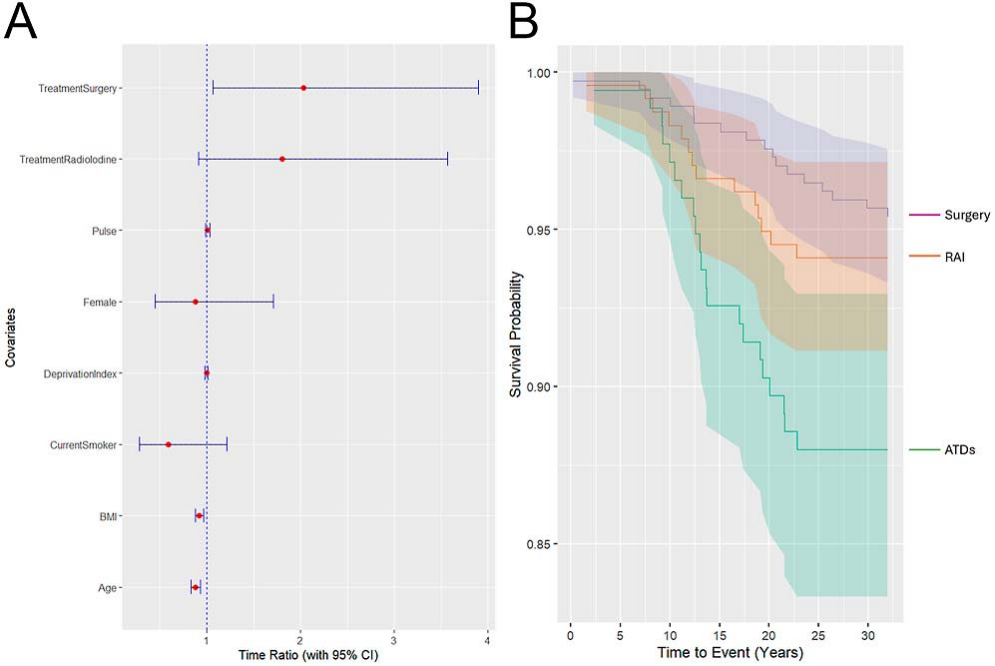
All-cause mortality following RAI and surgery using ATD treatment alone as the reference, including covariates. (A) Forest plot of time ratios with 95% CIs, constructed with an AFT model using a Weibull distribution. (B) Kaplan–Meier survival curve by treatment regimen (shaded areas represent 95% confidence intervals).

The results of the AFT survival analysis for all-cause mortality are summarised in Fig. 2. Treatment with ATDs was set as the reference, against which the other treatment regimens are measured. Participants treated with RAI showed a time ratio of 1.80 (95% CI 0.91–3.56) in time-to-event (all-cause mortality) relative to ATDs. A similar increase in the time ratio was shown for participants receiving surgical management (2.03, 95% CI 1.06–3.89). The percentages of right-censored data (i.e. where no date of all-cause mortality was available within the 30-year timeframe) are also shown in Table 4.

**Table 4:** Influence of treatment options on time ratios: all-cause mortality.

Treatment	Time ratio	95% CI	% right-censored data
ATD	Reference	Reference	88.0
RAI	1.80	0.91–3.56	94.1
Thyroidectomy	2.03	1.06–3.89	95.4

### Analysis of complications

**Figure 3:**
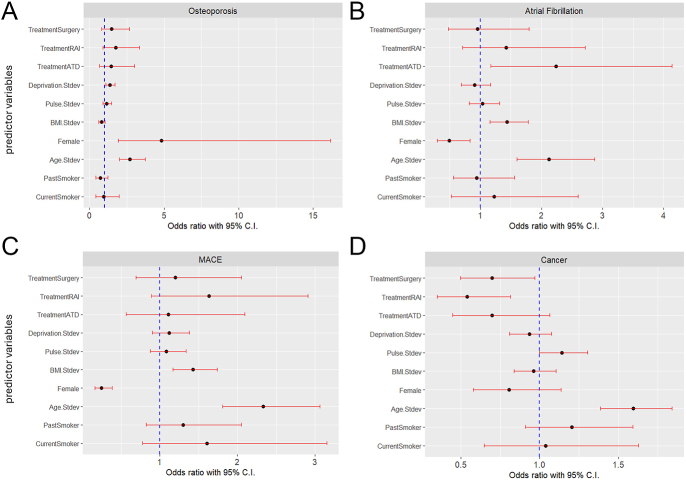
Forest plots for odds ratios of patients with hyperthyroidism subsequently being identified in the UK Biobank as presenting with comorbidities compared to a reference group of 793 matched controls. Treatments and covariates are shown in fixed order: (A) osteoporosis, (B) atrial fibrillation, (C) MACE, which represents selected cardiovascular events including strokes, myocardial infarctions and heart failures (D) cancer (all types). For continuous variables, the odds ratio is shown per standard deviation of the measure, not per unit of the measure, to allow for relative comparison of importance.

A series of comorbidity incidences in all participants with hyperthyroidism was reviewed (Fig. 3) based on the ICD-10 codes set out in methods. Adjusted odds ratios were calculated for atrial fibrillation and osteoporosis, two conditions that are known complications associated with hyperthyroidism. Other cardiovascular conditions were also investigated, specifically stroke, heart failure and myocardial infarction. These three were analysed as a group, with the adjusted odds ratio showing the incidence of one or more of these conditions in an individual. Cancer diagnoses were also included as another major comorbidity category.

These results are also shown numerically for the definitive treatments, as isolated predictor variables relative to ATDs, in Table 5.

**Table 5:** Influence of treatment options on adjusted odds ratio for outcomes associated with hyperthyroidism. Adjusted odds ratios (95% CI) vs control group.

	Osteoporosis	Atrial fibrillation	Selected CV events*	Cancer
Thyroidectomy	1.5 (0.8–2.7)	1.0 (0.5–1.8)	1.2 (0.7–2.1)	0.7 (0.5–1.0)
RAI	1.7 (0.9–3.3)	1.4 (0.7–2.7)	1.6 (0.9–2.9)	0.5 (0.3–0.8)
ATD	1.4 (0.6–3.0)	2.2 (1.2–4.1)	1.1 (0.6–2.1)	0.7 (0.4–1.1)

* Selected cardiovascular (CV) events include strokes, myocardial infarctions and heart failures (MACE).

### Peripheral blood biochemical analytes

UK Biobank data on peripheral blood biochemical analytes for individuals with hyperthyroidism and controls were also investigated post-treatment. These data represent a subset of the population of 1,586 analysed above, as not all of this group had blood biochemistry data available. Table 6 summarises the statistically significant pairwise differences, when FDR adjusted, alongside the mean values for each pair of treatment groups. Serum alkaline phosphatase showed the largest number of pairwise differences, with elevated concentrations for individuals treated by ATDs. Serum calcium was reduced for individuals treated by surgery and cystatin C concentrations were increased for individuals treated by ATDs. Other serum indicators (vitamin D, apolipoproteins, C-reactive protein, cholesterol, amino acids, direct bilirubin, glucose, total protein and triglycerides) did not show statistically significant differences between groups.

**Table 6:** Significant pairwise differences in peripheral blood biomarker analyte concentrations following treatment. *P*-values were calculated using the Wilcoxon rank sum test, with FDR correction by Benjamini–Hochberg. Indicators were tested but showed no FDR-corrected differences included alanine aminotransferase, albumin, apolipoprotein A, apolipoprotein B, aspartate aminotransferase, C-reactive protein, cholesterol, creatinine, direct bilirubin, gamma-glutamyltransferase, glucose, glycated haemoglobin (HbA1c), HDL cholesterol, IGF1, LDL direct, lipoprotein A, oestradiol, phosphate, rheumatoid factor, testosterone, total bilirubin, total protein, triglycerides, urate, urea and vitamin D.

Group 1	Group 2	*P* value	FDR *P* value	Mean value	
				Group 1	Group 2
Alkaline phosphatase					
Antithyroid medication	Healthy control	<0.001	<0.001	108.6 IU/L	84.3 IU/L
Antithyroid medication	Surgery	<0.001	<0.001	108.6 IU/L	83.9 IU/L
Antithyroid medication	Radioiodine	<0.001	0.006	108.6 IU/L	93.9 IU/L
Radioiodine	Healthy control	<0.001	0.006	93.9 IU/L	84.3 IU/L
Radioiodine	Surgery	<0.001	0.024	93.9 IU/L	83.9 IU/L
Calcium					
Surgery	Healthy control	<0.001	0.014	2.35 mMol/L	2.38 mMol/L
Cystatin C					
Antithyroid medication	Healthy control	<0.001	0.012	1.00 mg/L	0.88 mg/L

FDR, false discovery rate.

## Discussion

The main treatment options for hyperthyroidism are well-established, with three commonly used means of control: ATDs, RAI and thyroidectomy. Because each of the three has its own advantages and disadvantages, the preferred course of action for initial treatment has been a source of debate. NICE guidance in the UK changed in 2019 to a default approach of RAI as definitive treatment for most patients at their first presentation. In this work, UK Biobank data on patients treated for hyperthyroidism are analysed and compared to controls, which were matched to cases by sex and age. Survival analysis by AFT showed that RAI treatment was associated with a reduction in all-cause mortality measured by time ratio (time ratio 1.80, 95% CI 0.91–3.56), using ATDs as the reference treatment, albeit not quite reaching statistical significance. The same model showed thyroidectomy was also associated with a reduction in risk (time ratio 2.03, 95% CI 1.06–3.89). While confidence intervals were wide and the proportion of right-censored data high, these risk assessments were consistent with those previously reported in a study of the Taiwanese National Health Insurance Research Database, which also showed reductions in risk for RAI (hazard ratio 0.67, 95% CI 0.40–1.11) and thyroidectomy (hazard ratio 0.53, 95% CI 0.41–0.68), compared to ATD treatment (26). The results were also comparable with findings from the UK SAIL database, which found patients whose hyperthyroidism was resolved by RAI treatment experienced lower mortality relative to those treated with ATDs (hazard ratio 0.50, 95% CI 0.29–0.85) (27). Overall, these data reinforce previous findings that definitive treatments reduce risks of all-cause mortality relative to ATD treatment.

In addition to mortality, complications are also a relevant consideration for patients with hyperthyroidism and this was investigated here by calculation of adjusted odds ratios for diagnoses compared with the control group. For cardiovascular issues, risks generally remained elevated even after RAI treatment, albeit in many cases these results did not reach statistical significance. These raised odds ratios were in keeping with the EGRET study into the long-term mortality and cardiometabolic effects of hyperthyroidism treatments, which found increased risks of major cardiovascular events with RAI treatment (28). Risks were also elevated for atrial fibrillation, in particular for the ATD treated group (reaching statistical significance). Incidences of atrial fibrillation specifically under an ATD regimen are consistent with previous findings, likely reflecting episodes of uncontrolled/poorly controlled hyperthyroidism (29). Risks also remained elevated for all treatment types for osteoporosis. Regarding cancer, for all treatment types, diagnoses following treatment were less common than in controls. While some studies have indicated increased incidences of thyroid cancer associated with RAI (17, 18), the topic of induced cancer from treatment has been an ongoing source of debate (30). There is some evidence suggesting patients with hyperthyroidism have increased risks of cancer (31, 32), so logically treatment might reduce these risks, but the interactions between hyperthyroidism and cancer will be complex and vary by primary site. This naturally makes statistically powered investigations more challenging versus best practices (33, 34, 35).

Overall, these findings are concordant with other studies showing that in some cases thyroid-related abnormalities can be persistent even following effective antithyroid therapy (36). While confidence intervals were wide and frequency of events low, emphasising the need for caution in over-interpreting individual results, overall the findings shown here reinforce the importance of lifelong clinical monitoring of patients even after definitive treatment, especially for complications associated with hyperthyroidism such as cardiovascular and bone health.

Regarding peripheral blood biochemistry, UK Biobank data in the main were consistent with the previous literature observations. Increased alkaline phosphatase and cystatin C levels have been associated with hyperthyroidism (37, 38), and the group treated by ATDs showed statistically significant increases in these markers, especially compared with the other treatment regimens, where levels were closer to those in controls. Peripheral blood levels of calcium were slightly lower for patients treated by thyroidectomy, likely reflecting the surgical complication of hypoparathyroidism (39, 40). It should be noted, however, that while the result was statistically significant the effect size was small and median levels were still above the threshold of 2.20 mmol/L or lower for hypocalcaemia, suggestive that over the longer term cases of post-surgical hypoparathyroidism are mild and that supplementation regimens are effective in correcting hypocalcaemia. These data do not, however, capture short-term responses to treatments, given that the data for treatments come from various health registers and timepoints and UK Biobank recruitment/baseline measurement followed the dates of initial treatment by an average of 10 years.

This work does include a number of other limitations. UK Biobank data are not fully representative of the UK population or indeed wider global populations (41). While several potential confounders were included in the statistical modelling (adjusted odds ratio, AFTs), it is important to note that the UK Biobank is an observational retrospective dataset. Given the wide confidence intervals, even a large study cohort as offered by the UK Biobank may have lacked statistical power compared to a targeted study (e.g. survival analysis data in the UK Biobank lack sufficient events at this time). Features such as higher levels of deprivation in participants with hyperthyroidism were consistent with previous findings of a positive association in the UK between deprivation and autoimmune disorders including GD, but biases in hyperthyroidism recruitment cannot be ruled out (42). The lack of information concerning detailed aetiology of hyperthyroidism and the lack of serum thyroid hormone measurements in the UK Biobank create additional challenges for the identification of phenotypically different subgroups with differing risk profiles (43). For example, elevated pulse rate in patients treated by ATD may be driven by treatment differences or may be due to fitter patients being selected for RAI or thyroidectomy. For ATD treatment, UK Biobank data lacked prescription dates and information on drug dosages and frequency of use, limiting comparisons of the risks associated with ATDs to those associated with RAI or thyroidectomy. Issues such as the lower count of ATD treated patients/high number of patients with no apparent treatment regimen may also signify some recall bias in the population, inconsistent accuracy of data records in different settings or coding errors. In addition, as noted by NICE, there may be specific clusters of patients that are phenotypically different and might not respond well to specific treatments. Such issues are difficult to address with retrospective analyses and would be better investigated in future prospective work. Finally, the Biobank data do not contain wellbeing data so that although we could examine hard outcomes, we had no insight into how patients were feeling following each of these treatment options (44).

In conclusion, this work finds little evidence of increased risks related to RAI and indeed based on survival analysis, ATD appears to be the highest risk option for treatment in terms of all-cause mortality. This is supportive of NICE changes in guidance towards RAI for definitive first-line treatment. Nonetheless, the data are additionally consistent with other findings that RAI treatment (or any treatment) does not fully eliminate risks of complications related to hyperthyroidism. This reinforces the need for both clear communication where there may be increased risks of conditions such as osteoporosis as well as clinical support for patients even after definitive treatment.

## Declaration of interest

The authors declare that there is no conflict of interest that could be perceived as prejudicing the impartiality of the work reported.

## Funding

This work did not receive any specific grant from any funding agency in the public, commercial or not-for-profit sector.

## Author contribution statement

MS and NG were responsible for conceptualization. MS was responsible for methodology. MS and KE handled the software. MS and KE performed the formal analysis. MS and KE conducted the investigation. NG provided the resources. KE managed data curation. MS and KE prepared the original draft. NG, SP and EG were responsible for review and editing. MS and KE produced the visualizations. SP and NG provided supervision. MS and NG managed project administration. All authors have read and agreed to the published version of the manuscript.

## Data availability

UK Biobank data are available to registered researchers; the underlying data may only be accessed through the Biobank’s Research Analysis Platform. More information on data access is available at https://www.ukbiobank.ac.uk/enable-your-research/about-our-data [accessed 4 December 2024].
